# Supplementary material for: Genome-Wide Identification and Expression Profiling Analysis of WOX Family Protein-Encoded Genes in Triticeae Species
Source: Int J Mol Sci. 2021 Aug 28;22(17):9325. doi: 10.3390/ijms22179325 (PMC8431079; doi:10.3390/ijms22179325)
Supplement: Supplementary file 1 [file ijms-22-09325-s001.zip › ijms-1285515-supplementary.pdf]

International Journal of *Molecular Sciences*

MDPI

Supplementary Materials

Genome-wide identification and expression profiling analysis of WOX family proteins encoded genes in Triticeae plant species

Lei Shi[1,2], Ke Wang[1], Lipu Du[1], Yuxia Song[2], Huihui Li[1]* and Xingguo Ye[1,3,*]

1 Institute of Crop Sciences, Chinese Academy of Agricultural Sciences, Beijing 100081, China. s5266@126.com (L.S.); wangke03@caas.cn (K.W.); dulipu@caas.cn (L.D.); lihuihui@caas.cn (H.L.); yexingguo@caas.cn (X. Y.)
2 Key Laboratory of Agricultural Biotechnology of Ningxia, Ningxia Academy of Agriculture and Forestry Sciences, Yinchuan 750002, China. songyx666@163.com (Y.S.)
3 National Key Facility of Crop Gene Resources and Genetic Improvement, Chinese Academy of Agricultural Sciences, Beijing 100081, China. yexingguo@caas.cn (X. Y.)
* Correspondence: yexingguo@caas.cn (X. Y.).

International Journal of
Molecular Sciences

MDPI

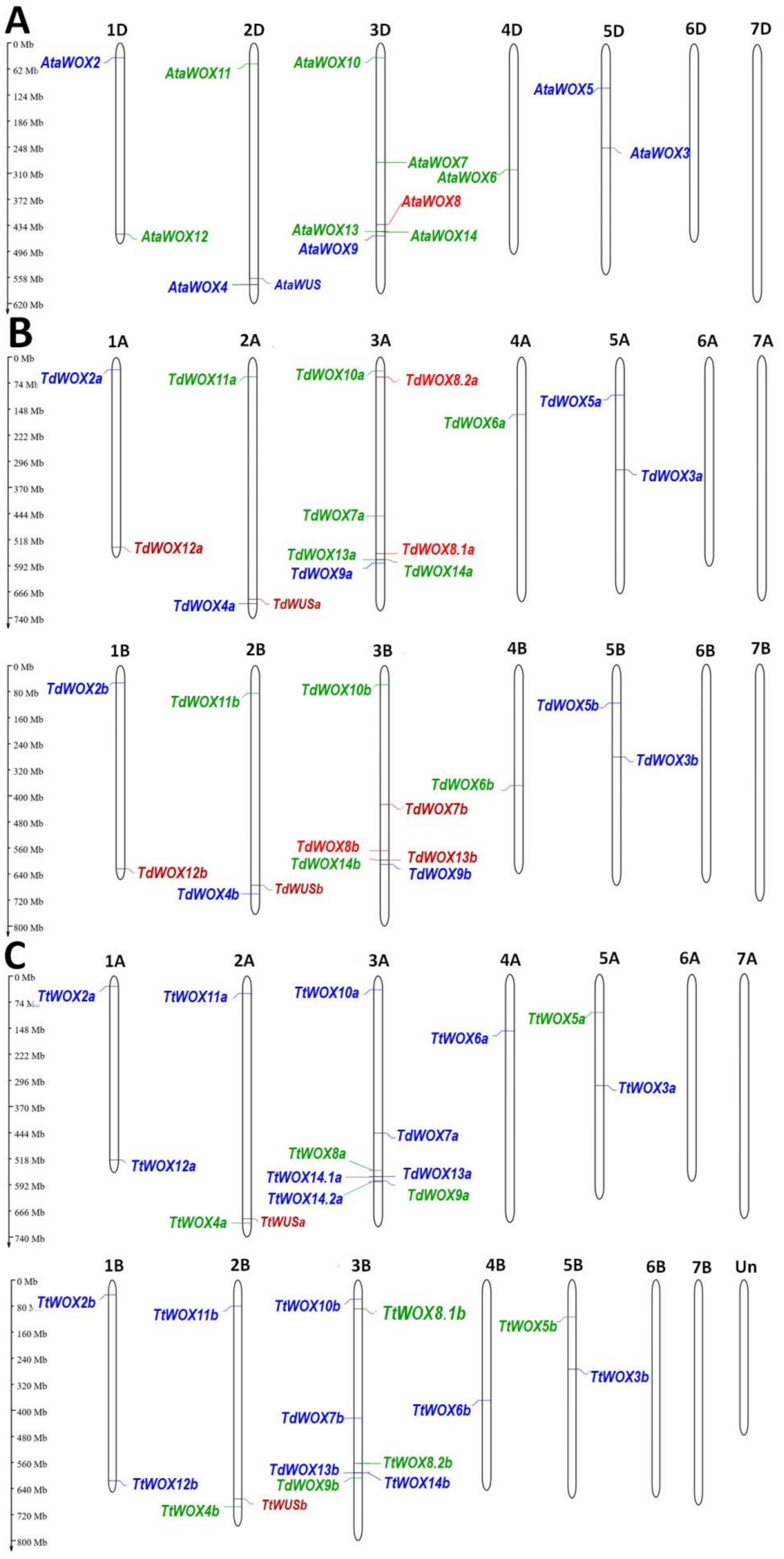

International Journal of
Molecular Sciences

MDPI

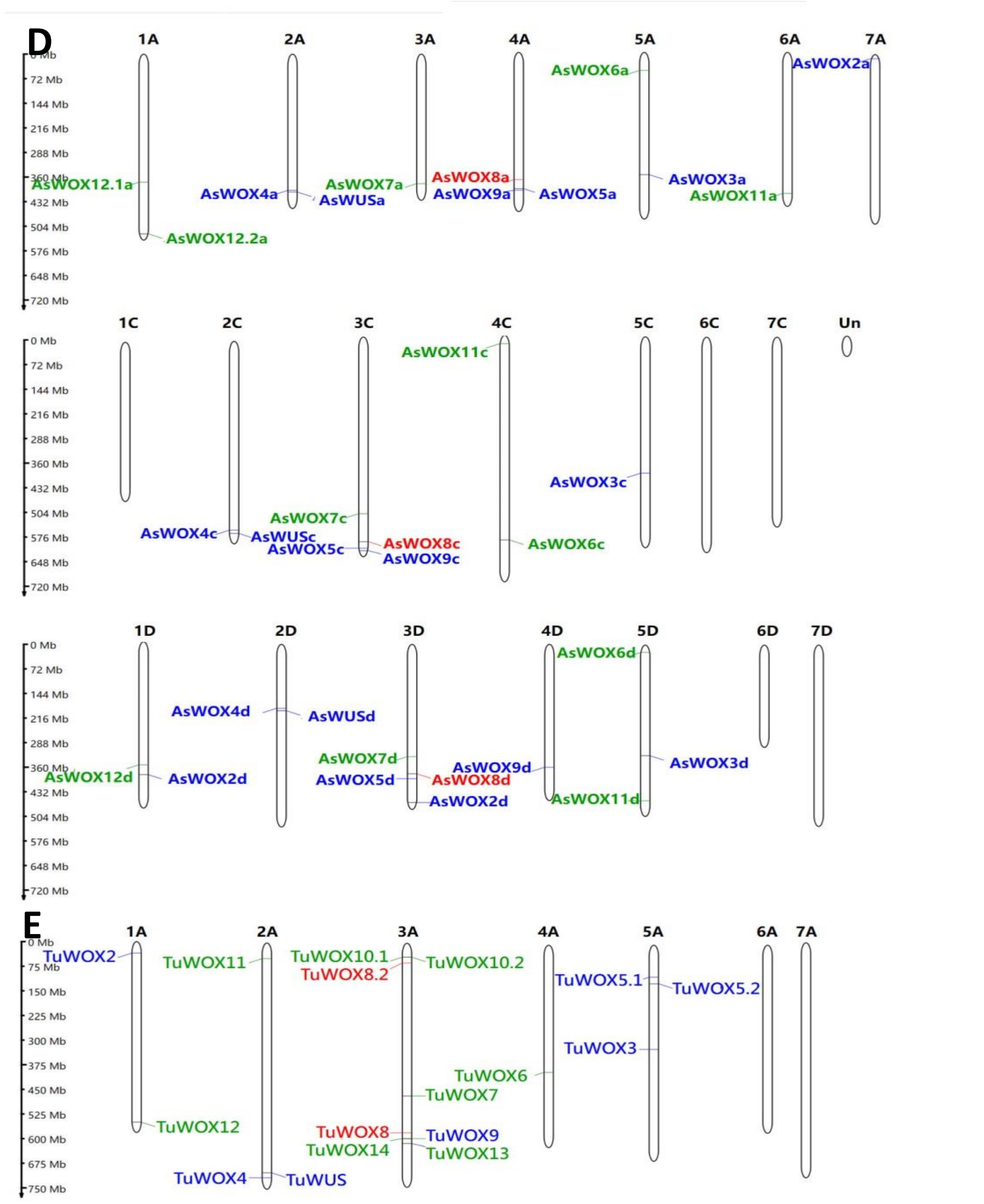

Supplementary Material 1: Figure S1. Chromosomal location of *WOX* genes in *A. tauschii*, *T. dicoccoides*, *T. turgidum* , *A. sativa*, and *T. urartu*.

Chromosomal location of *WOX* genes in *A. tauschii* (A), *T. dicoccoides* (B), *T. turgidum* (C), *A. sativa* (D) and *T. urartu* (E). Vertical coordinate displayed length of chromosomes, and the *WOX* members in WUS clade were shown as blue type, the *WOX* members in intermediate clade were shown as green type, the *WOX* members in ancient clade were shown as red type.

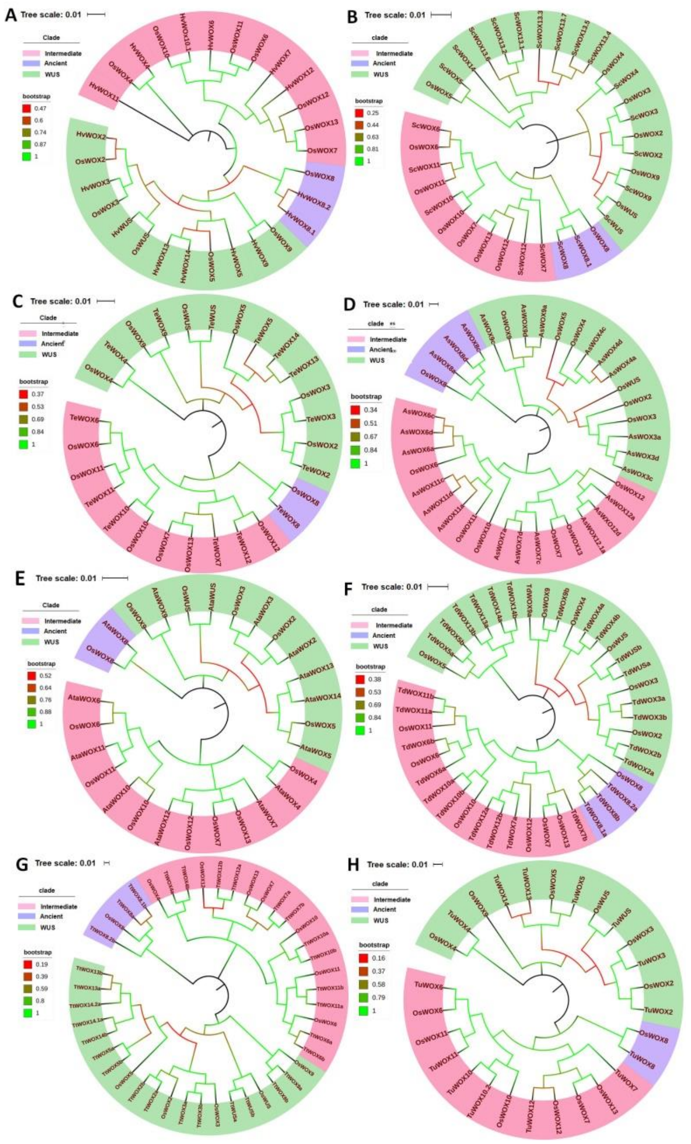

Supplementary Material 2: Figure S2. Phylogenetic relationships of WOX proteins between *H. vulgare*, *S. cereal*, *T. elongatum*, *A. sativa*, *T. dicoccoides*, *T. turgidum*, *A. tauschii* or *T. urartu* and *O. sativa*.

A. Phylogenetic tree derived from multiple sequence alignment generated with entire OsWOX and HvWOX protein sequences; B. Phylogenetic tree derived from OsWOX and ScWOX protein sequences; C. Phylogenetic tree derived from OsWOX and TeWOX protein sequences; D. Phylogenetic tree derived from OsWOX and AsWOX protein sequences; E. Phylogenetic tree derived from OsWOX and AtaWOX protein sequences; F. Phylogenetic tree derived from OsWOX and TdWOX protein sequences; G. Phylogenetic tree derived from OsWOX and TtWOX protein sequences; H. Phylogenetic tree derived from OsWOX and TuWOX protein sequences. Phylogenetic tree was constructed based on the sequences of WOX proteins in *O. sativa*, *H. vulgare*, *T. turgidum*, *T. dicoccoides*, *A. tauschii* and *T. urartu*, performed by the MEGA X using maximum likelihood approach and 1000 bootstrap replicates. WUS clade, intermediate clade and ancient clade were labeled in outside circle and displayed in different colored ranges. Scale plate and legend in upper left displayed tree scale and bootstrap value.

International Journal of
Molecular Sciences

MDPI

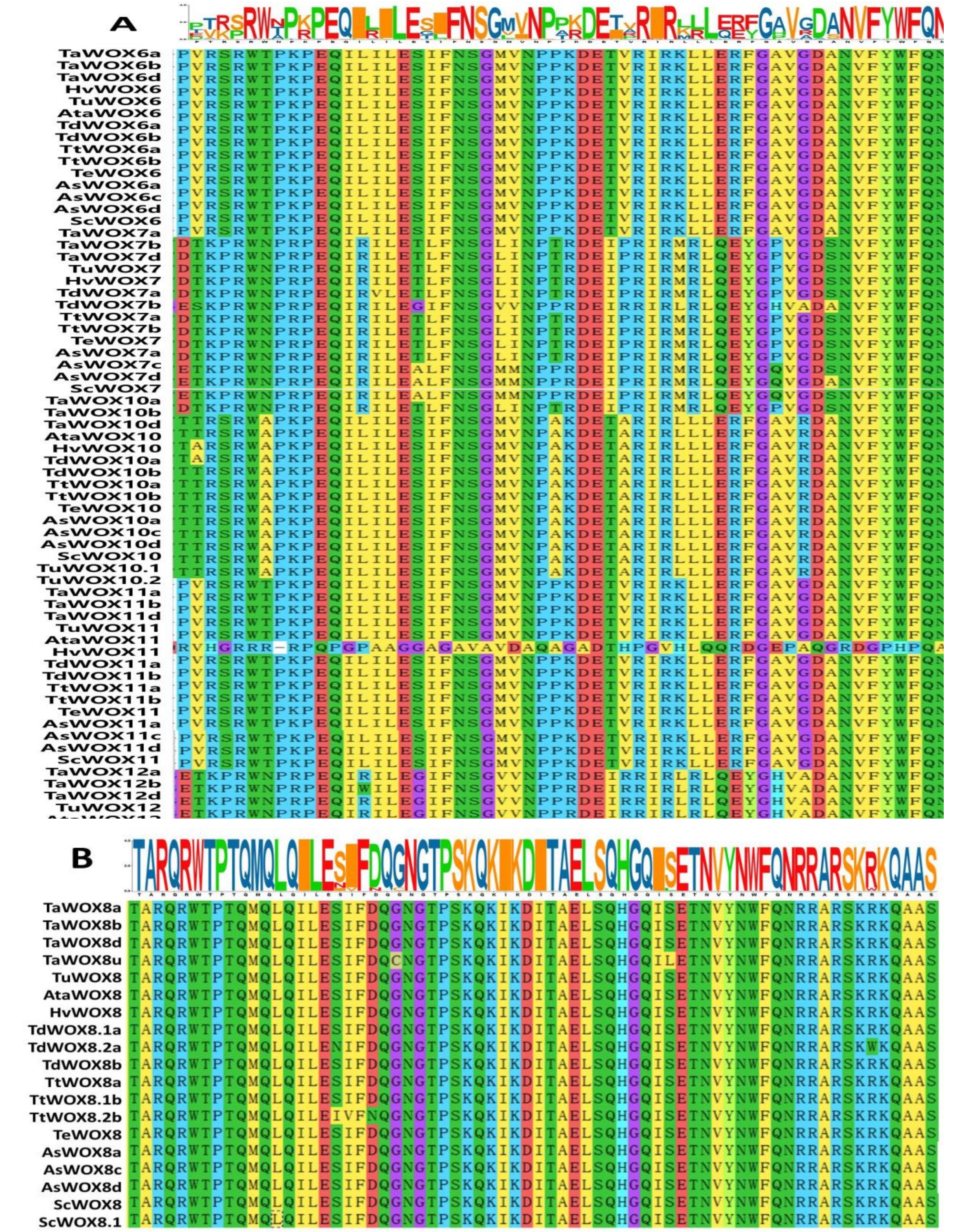

Supplementary Material 3: Figure S3. Alignment of WOX homeodomains from intermediate and acient clade of nine Triticeae species.

Phylogenetic alignment of homeodomain sequences was conducted by ClustalW algorithm using MEGA X. The LOGO of protein sequences represents the relative frequency of an amino acid at this position and the content of the aligned sequences at a position in bit (max. 4.322 bit for proteins, i.e. log220). A. Multiple sequence alignment among the HOX homeodomain of all intermediate clade WOX proteins in the nine *Triticeae* species. B. Multiple sequence alignment among the HOX homeodomain of all ancient clade WOX proteins in these nine Triticeae species.

International Journal of *Molecular Sciences*

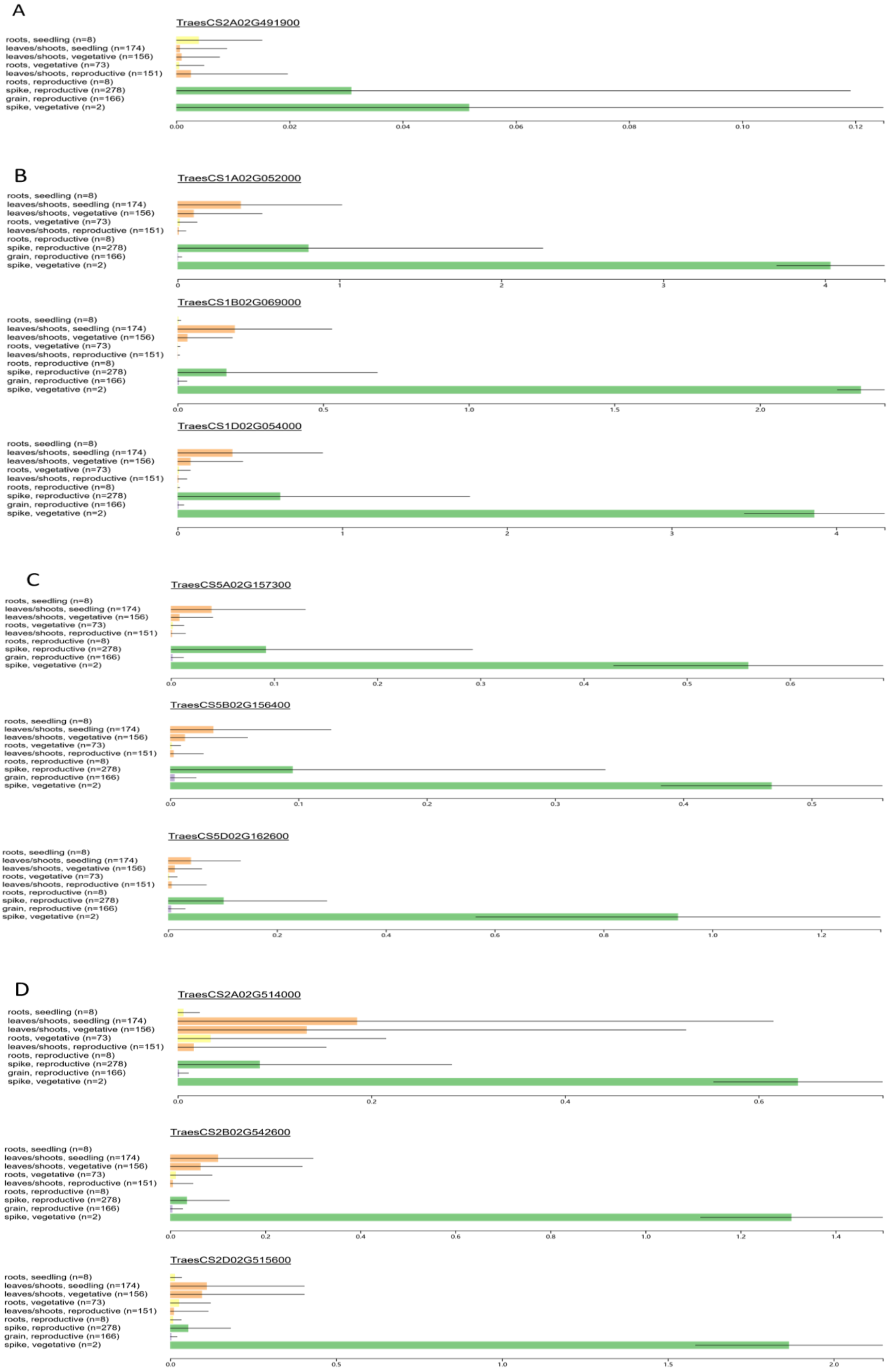

International Journal of
Molecular Sciences

MDPI

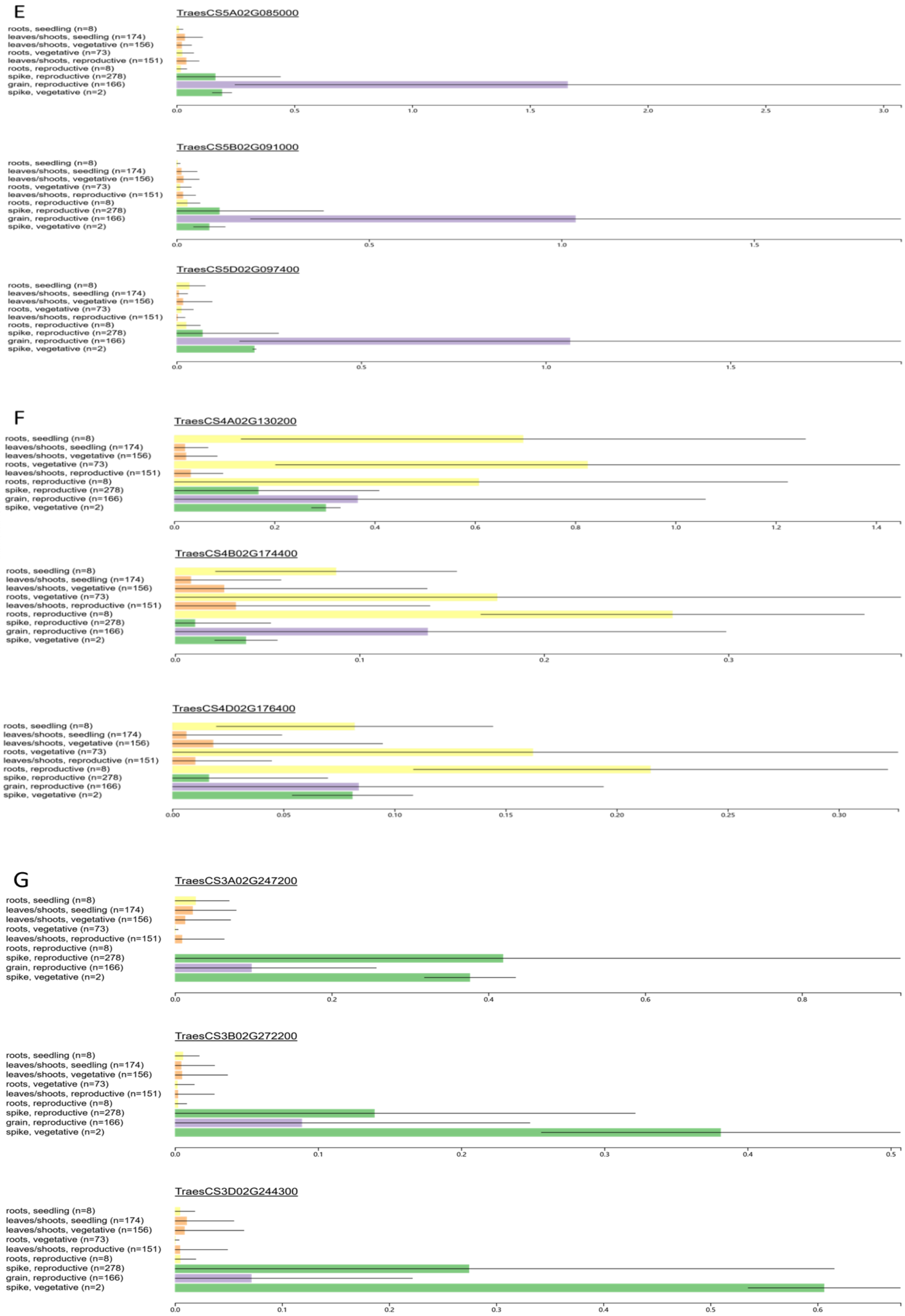

International Journal of *Molecular Sciences*

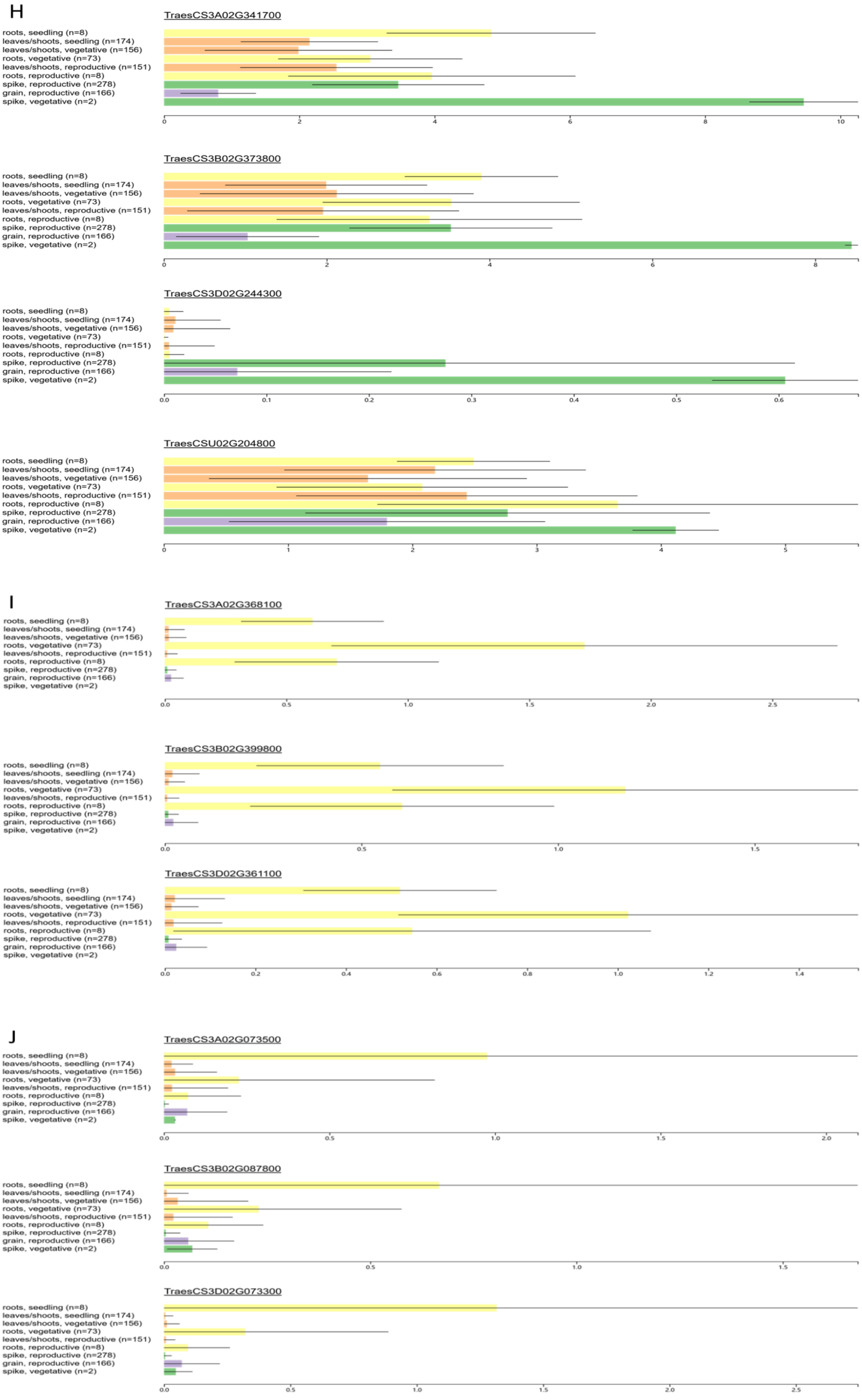

International Journal of
Molecular Sciences

MDPI

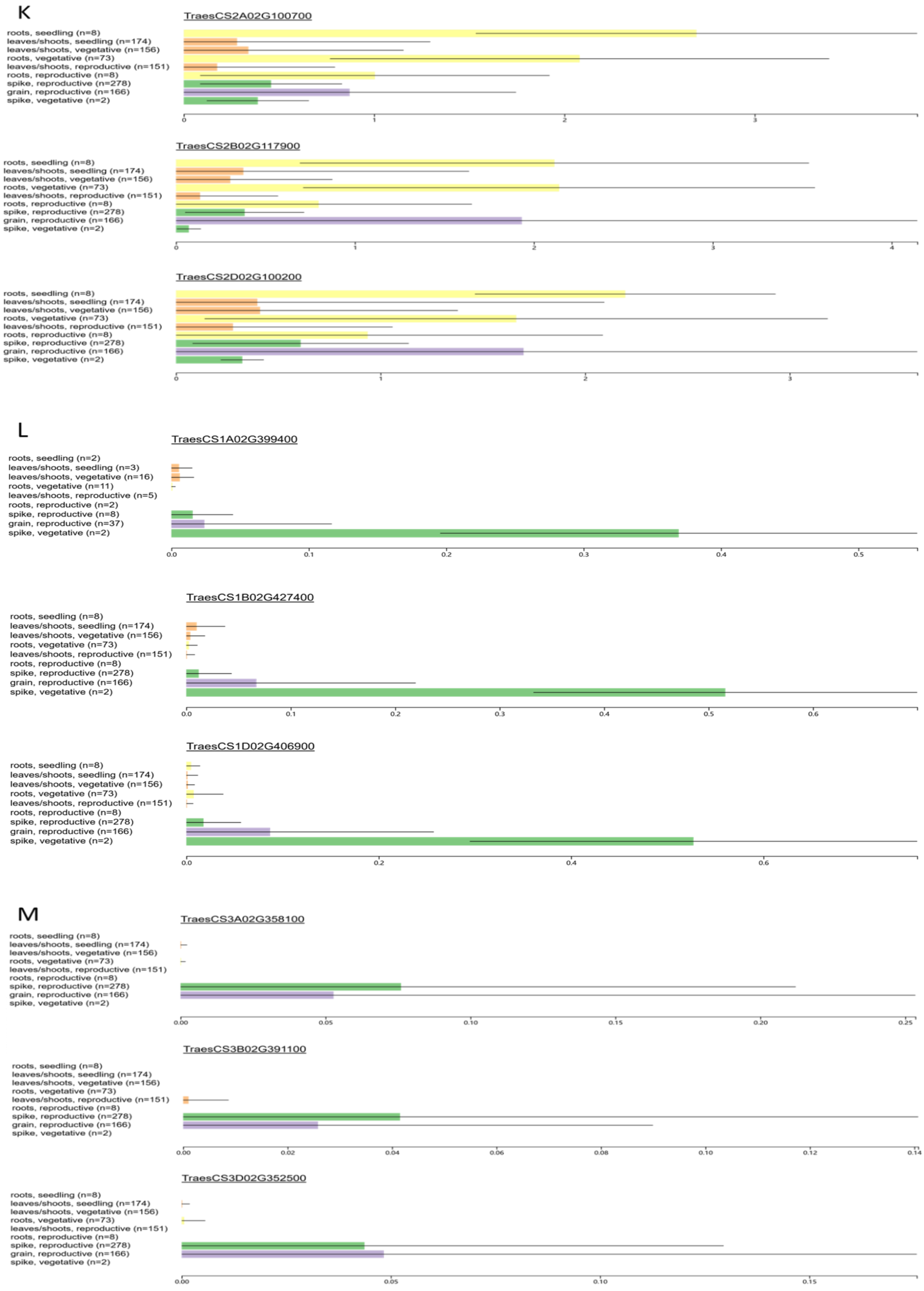

International Journal of
Molecular Sciences

MDPI

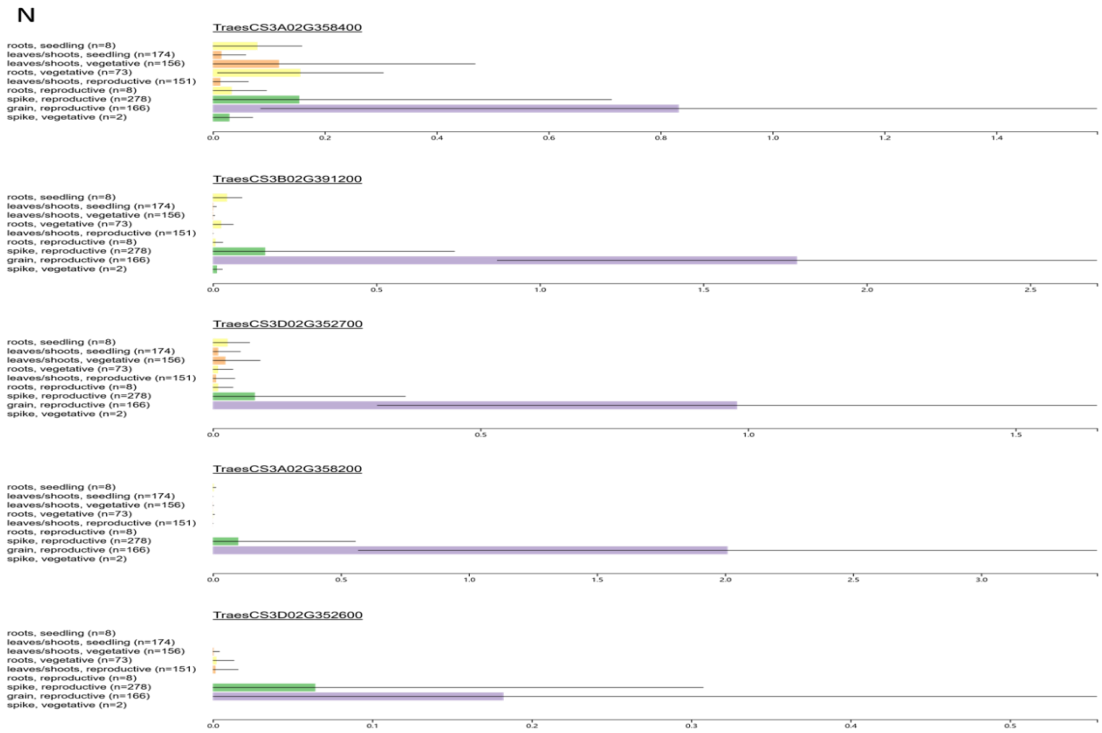

Supplementary Material 4: Figure S4. Expression profiling of *TaWOX* genes in various organs at different stages in wheat.

Data is downloaded from expVIP website (http://wheat-expression.com). A: *TaWUS*; B: *TaWOX2*; C: *TaWOX3*; D:*TaWOX4*; E: *TaWOX5*; F: *TaWOX6*; G: *TaWOX7*; H: *TaWOX8*; I: *TaWOX9*; J: *TaWOX10*; K: *TaWOX11*; L: *TaWOX12*; M: *TaWOX13*; N: *TaWOX14*. Vertical coordinate refers to organs, stages, and sample number; horizontal ordinate refers to relative expression quantity of target genes.

International Journal of *Molecular Sciences*

MDPI

Supplementary Material 5: Table S1. Summary of the AtWOX and OsWOX gene family members

Gene	Gene locus	Genbank ID of mRNA	Genbank ID of protein	Uniprot ID
AtWUS	*AT2G17950*	NM_127349.4	NP_565429.1	Q9SB92
AtWOX1	*AT3G18010*	NM_112682.4	NP_188428.3	Q6X7K0
AtWOX2	*AT5G59340*	NM_125325.3	NP_200742.2	Q6X7K1
AtWOX3	*AT2G28610*	NM_128422.3	NP_180429.1	Q9SIB4
AtWOX4	*At1G46480*	NM_103605.7	NP_175145.2	Q6X7J9
AtWOX5	*At3G11260*	NM_111961.4	NP_187735.2	Q8H1D2
AtWOX6	*AT2G01500*	NM_126211.2	NP_565263.1	Q9ZVF5
AtWOX7	*At5G05770*	NM_120659.2	NP_196196.1	Q9FFK0
AtWOX8	*AT5G45980*	NM_123966.3	NP_199410.2	Q6X7J5
AtWOX9	*AT2G33880*	NM_128948.4	NP_180944.2	Q6X7J4
AtWOX10	*AT1G20710*	NM_101923.1	NP_173494.1	Q9LM83
AtWOX11	*AT3G03660*	NM_111237.3	NP_187016.2	Q6X7J3
AtWOX12	*AT5G17810*	NM_121787.3	NP_197283.2	Q8GY25
AtWOX13	*AT4G35550*	NM_119720.4	NP_195280.1	O81788
AtWOX14	*AT1G20700*	NM_101922.3	NP_173493.2	Q9LM84
OsWUS	*Os04g0663600*	XM_015779885.2	XP_015635371.1	Q7XM13
OsWOX2	*Os05g0118700*	XM_015783597.2	XP_015639083.1	Q5W7C3
OsWOX3	*Os11g0102100/ Os12g0101600*	XM_015761276.2	XP_015616762.1	Q33DK1
OsWOX4	*Os04g0649400*	XM_015779881.2	XP_015635367.1	Q7XTV3
OsWOX5	*Os01g0840300*	XM_015788740.2	XP_015644226.1	Q8LR86
OsWOX6	*Os03g0325600*	XM_015773520.2	XP_015629006.1	Q10M29
OsWOX7	*Os01g0667400*	XM_026019881.1	XP_025875666.1	Q0JKK6
OsWOX8	*Os01g0818400*	XM_015770969.2	XP_015626455.1	Q5QMM3
OsWOX9	*Os01g0854500*	XM_015787499.2	XP_015642985.1	Q8W0F1
OsWOX10	*Os08g0242400*	XM_015794156.1	XP_015649642.1	Q6Z3L4
OsWOX11	*Os07g0684900*	XM_015790233.2	XP_015645719.1	Q0D3I7
OsWOX12	*Os05g0564500*	XM_026025403.1	XP_025881188.1	A3B6V0
OsWOX13	*Os07g0533100*	XM_015790527.2	XP_015646013.1	A3BKM2

International Journal of *Molecular Sciences*

MDPI

Supplementary Material 6: Table S2. Characteristics of *AtaWOX* gene family members in *A. tauschii*

Gene	Gene locus	Chromosome	Gene stretch region	mRNA length (bp)	Protein sequence length (aa)	Uniprot ID	RefSeq ID
AtaWOX2	*AET1Gv20131800*	1D	36,944,248-36,945,654:-1	1256	243	R7VZP3	XM_020308608.1
AtaWOX12	*AET1Gv20953700*	1D	477,482,405-477,487,246:1	1750	461	N1QTU6	XM_020313635.1
AtaWOX11	*AET2Gv20201700*	2D	53,152,025-53,154,730:1	1402	285	A0A453AN31	XM_020326401.1
AtaWOX4	*AET2Gv21131600*	2D	604,547,042-604,548,705:1	1432	235	M8BW89	XM_020306027.1
AtaWOX10	*AET3Gv20162600*	3D	36,379,013-36,380,597:1	1250	261	M8C4V1	XM_020344627.1
AtaWOX7	*AET3Gv20594700*	3D	346,711,567-346,714,983:-1	1542	513	M8BNX7	XM_020303677.1
AtaWOX8	*AET3Gv20773400*	3D	455,036,105-455,039,875:1	1630	262	M8CAA6	XM_020332450.1
AtaWOX13	*AET3Gv20805500*	3D	471,027,549-471,091,495:-1	1156	285	R7W9P1	
AtaWOX14	*AET3Gv20805900*	3D	471,208,547-471,212,126:-1	1272	291	N1QRI3	
AtaWOX9	*AET3Gv20824700*	3D	482,688,233-482,689,713:-1	1271	210	M8BYV0	
AtaWOX6	*AET4Gv20460500*	4D	314,146,370-314,149,574:1	1000	314	M8CH34	XM_020309078.1
AtaWOX5	*AET5Gv20233200*	5D	111,775,850-111,778,228:1	969	322	A0A023R759	XM_020308947.1
AtaWOX3	*AET5Gv20399900*	5D	260,643,657-260,644,829:1	1026	242	R7W601	XM_020339660.1
AtaWUS		2D	588,432,717-588,433,925:1	927	308		

International Journal of Molecular Sciences

MDPI

Supplementary Material 7: Table S3. Characteristics of *TdWOX* gene family members in *T. dicoccoides*

Gene	Gene locus	Chromosome	Gene stretch region	mRNA length (bp)	Protein sequence length (aa)
TdWOX2a	*TRIDC1AG007150*	1A	35,225,067-35,226,474:-1	1267	263
TdWOX12a		1A	562,999,481-563,003,353	1461	486
TdWOX2b	*TRIDC1BG009160*	1B	56,235,853-56,237,613:-1	1514	264
TdWOX12b		1B	655,616,838-655,620,923	1458	485
TdWOX11a	*TRIDC2AG011820*	2A	56,306,448-56,309,162:1	854	262
TdWOX4a	*TRIDC2AG072250*	2A	731,229,384-731,230,228:1	690	229
TdWOX11b	*TRIDC2BG014670*	2B	89,285,280-89,288,104:1	864	260
TdWOX4b	*TRIDC2BG078290*	2B	736,558,472-736,559,499:-1	699	232
TdWOX10a	*TRIDC3AG008680*	3A	42,317,316-42,318,397:1	810	193
TdWOX8.2a	*TRIDC3AG012070*	3A	60,093,235-60,099,789:-1	1707	172
TdWOX7a	*TRIDC3AG037080*	3A	472,489,137-472,490,767:1	1533	510
TdWOX8.1a	*TRIDC3AG049280*	3A	584,617,832-584,621,605:1	1046	261
TdWOX13a	*TRIDC3AG051560*	3A	602,962,138-602,964,193:-1	2042	181
TdWOX14a	*TRIDC3AG051570*	3A	603,033,097-603,086,787:-1	1166	290
TdWOX9a	*TRIDC3AG052920*	3A	613,614,879-613,616,278:-1	643	214
TdWOX10b	*TRIDC3BG011840*	3B	62,220,403-62,221,372:-1	749	213
TdWOX7b		3B	447,470,878-447,473,971:-1	1548	515
TdWOX8b	*TRIDC3BG055520*	3B	597,159,634-597,163,486:1	1513	261
TdWOX13b		3B	627,499,895-627,501,751:-1	900	299
TdWOX14b	*TRIDC3BG058280*	3B	627,719,369-627,721,911:-1	1198	290
TdWOX9b	*TRIDC3BG059490*	3B	641,913,687-641,914,697:-1	636	211
TdWOX6a	*TRIDC4AG018040*	4A	170,474,653-170,477,215:-1	1001	315
TdWOX6b	*TRIDC4BG031600*	4B	389,709,929-389,712,462:1	1009	317
TdWOX5a	*TRIDC5AG013020*	5A	111,615,636-111,617,548:1	953	318
TdWOX3a	*TRIDC5AG026090*	5A	334,542,650-334,543,892:1	1095	237

TdWOX5b	*TRIDC5BG014980*	5B	122,875,190-122,877,486:1	966	321
TdWOX3b	*TRIDC5BG027470*	5B	297,256,041-297,257,278:-1	1103	241
TdWUSa		2A	717,371,085-717,372,278:1	927	308
TdWUSb		2B	709,748,669-709,749,875:1	936	311

International Journal of Molecular Sciences

MDPI

Supplementary Material 8: Table S4. Characteristics of *TtWOX* gene family members in *T. turgidum*

Gene	Gene locus	Chromosome	Gene stretch region	mRNA length (bp)	Protein sequence length (aa)
TtWOX2a	*TRITD1Av1G014400*	1A	30,266,253-30,267,185:-1	792	263
TtWOX12a	*TRITD1Av1G210580*	1A	548,095,711-548,099,750:-1	1461	486
TtWOX2b	*TRITD1Bv1G018490*	1B	47,977,816-47,978,741:-1	795	264
TtWOX12b	*TRITD1Bv1G213780*	1B	645,322,215-645,325,522:1	1458	485
TtWOX11a	*TRITD2Av1G026520*	2A	52,846,448-52,848,548:1	798	265
TtWOX4a	*TRITD2Av1G275690*	2A	735,363,729-735,364,662:1	705	234
TtWOX11b	*TRITD2Bv1G034440*	2B	83,084,542-83,086,937:1	786	261
TtWOX4b	*TRITD2Bv1G239870*	2B	726,776,414-726,777,497:-1	714	237
TtWOX10a	*TRITD3Av1G020310*	3A	41,781,579-41,782,652:1	807	268
TtWOX7a	*TRITD3Av1G165910*	3A	468,275,958-468,279,104:1	1548	515
TtWOX8a	*TRITD3Av1G210120*	3A	580,017,802-580,022,111:1	798	265
TtWOX13a	*TRITD3Av1G217260*	3A	597,915,150-597,916,973:-1	906	301
TtWOX14.2a	*TRITD3Av1G217280*	3A	597,987,122-597,990,043:-1	867	288
TtWOX9a	*TRITD3Av1G222700*	3A	610,880,586-610,883,548:1	639	212
TtWOX14.1a	*TRITD3Av1G223690*	3A	613,842,920-613,845,442:-1	873	290
TtWOX10b	*TRITD3Bv1G023880*	3B	62,602,349-62,603,796:1	837	278
TtWOX8.2b	*TRITD3Bv1G035130*	3B	90,684,051-90,690,161:-1	1608	535
TtWOX7b	*TRITD3Bv1G141830*	3B	442,112,979-442,116,074:-1	1548	515
TtWOX8.1b	*TRITD3Bv1G192740*	3B	588,758,146-588,761,021:-1	786	261
TtWOX13b	*TRITD3Bv1G202750*	3B	620,225,076-620,226,932:-1	900	299
TtWOX14b	*TRITD3Bv1G202810*	3B	620,444,973-620,447,190:-1	873	290
TtWOX9b	*TRITD3Bv1G207240*	3B	634,976,698-634,977,442:-1	630	209
TtWOX6a	*TRITD4Av1G066630*	4A	168,111,221-168,113,757:-1	948	315
TtWOX6b	*TRITD4Bv1G109220*	4B	386,507,631-386,510,136:1	954	317
TtWOX5a	*TRITD5Av1G044880*	5A	113,122,758-113,124,660:1	957	318

International Journal of *Molecular Sciences*

TtWOX3a	*TRITD5Av1G117410*	5A	330,291,871-330,292,732:1	726	241
TtWOX5b	*TRITD5Bv1G042650*	5B	118,715,492-118,717,394:1	966	321
TtWOX3b	*TRITD5Bv1G098090*	5B	288,212,824-288,213,684:-1	726	241
TtWUSa		2A	721,813,175-721,814,364:1	927	308
TtWUSb		2B	702,358,638-702,359,840:1	949	314

International Journal of Molecular Sciences

MDPI

Supplementary Material 9: Table S5. Characteristics of *TuWOX* gene family members in *T. urartu*

Gene	Gene locus	Chromosome	Gene stretch region	mRNA length (bp)	Protein sequence length (aa)	Uniprot ID
TuWUS		2A	703254702-703255899:1	927	308	
TuWOX 2	*TRIUR3_16981*	1A	35836123-35837055:-1	489	162	M8AVP1
TuWOX3		5A	318694678-318695541:1	726	241	
TuWOX 4	*TRIUR3_10705*	2A	717747572-717748508:1	702	233	M7ZDD1
TuWOX5.1		5A	97212511-97214413:1	957	318	
TuWOX5.2		5A	117140992-117142894:1	957	318	
TuWOX6	*TRIUR3_34808*	4A	389952598-389954998:-1	924	307	M7ZHX8
TuWOX7		3A	466301882-466304977:1	1548	515	
TuWOX8	*TRIUR3_23636*	3A	581099107-581101923:1	798	265	M7YSU5
TuWOX 8.2	*TRIUR3_03990*	3A	59810323-59815510:-1			M7YTI6
TuWOX 9	*TRIUR3_26391*	3A	208,605-211,568:-1	702	233	M7ZY58
TuWOX10.1	*TRIUR3_34876*	3A	41611617-41612472:1	783	260	M7ZJ11
TuWOX10.2		3A	42098538-42099493:1	783	260	
TuWOX11		2A	47359405-47361393:1	798	265	
TuWOX12		1A	552324202-552328073:1	1461	486	
TuWOX 13	*TRIUR3_08856*	3A	53,497-55,135:1	723	240	M7ZSR7
TuWOX 14		3A	598240782-598243297:-1	873	290	

International Journal of *Molecular Sciences*

MDPI

Supplementary Material 10: Table S6. Primers for qPCR amplification

Primer name	Primer sequence
q*TaWUS*F	5'-GACAAGCAGAGCGTCATGTGG-3'
q*TaWUS*R	5'-AGTAGAGGTCCTTGAGGATCTTGAC-3'
q*TaWOX2*F	5'-GCTGATGATCCTGGAGGAGATGTAC-3'
q*TaWOX2*R	5'-AAGACGTTCTTGCCCTCGATGC-3'
q*TaWOX3*F	5'-ATCACCACCACACATACGCTACC-3'
q*TaWOX3*R	5'-TGTTGTTAATGTTGCTGTTGTCCA-3'
q*TaWOX4*F	5'-AGATCAAGGTGCTGGAGGCGCTG-3'
q*TaWOX4*R	5'-GAAGACGTTCTTGCCCTCGATCC-3'
q*TaWOX5*F	5'-ACAGGCCACCATGTACCACCA-3'
q*TaWOX5*R	5'-GGGAACAGGTTCAGGGTCTCG-3'
q*TaWOX6*F	5'-CTCGTCGTCCTCGTCTGGAAGCAC-3'
q*TaWOX6*R	5'-GGCTCCCAACGAATCCCATCTGA-3'
q*TaWOX7*F	5'-TCTTCTACTGGTTCCAGAACCGCA-3'
q*TaWOX7*R	5'-GATGGAACTGCTTGGGAGGTGG-3'
q*TaWOX8*F	5'-CAGGTATGAGGCTTGGTAATC-3'
q*TaWOX8*R	5'-CTGAAATCTGTCCGTGCTG-3'
q*TaWOX9*F	5'-GAACGTCTTCTACTGGTTCCAGAAC-3'
q*TaWOX9*R	5'-TGTAGCCTCTGGCCTCACGCTT-3'
q*TaWOX10*F	5'-GTGCCCGCCAGCTCCAGCAGTC-3'
q*TaWOX10*R	5'-GGCGTCGTCTAACAAGAAACGG-3'
q*TaWOX11*F	5'-GGAGTCCATCTTCAACAGCGGCAT-3'
q*TaWOX11*R	5'-GGAACCAGTAGAAGACGTTGGCGT-3'
Q*TaWOX12*F	5'-GAGCAACACCCACACCAGATCCT-3'
q*TaWOX12*R	5'-GTTCTTGGTGCGAGACTTGCGGT-3'
q*TaWOX13*F	5'-AGCCTACTACTCGCAGATGCA-3'
q*TaWOX13*R	5'-CTGTTGTTGGTCGAAGGAAAC-3'
q*TaWOX14*F	5'-CTGTCCCTTTCCACTCCCCGTC-3'
q*TaWOX14*R	5'-CGCGATCACCTGATGTATCTCCTC-3'
q*TaActin*F	5'-TGTTGTTCTCAGTGGAGGTTCT-3'
q*TaActin*R	5'-CTGTATTTCCTTTCAGGTGGTG-3'

International Journal of *Molecular Sciences*

MDPI

Supplementary Material 11: Table S7. Primers for PCR amplification

Primer name	Primer sequence
*WUSa*F	5’- CACGTCCTCTCTCTCTCTCTCG-3’
*WUSa*R	5’- GGTGCTGTCTCCCCAGCAAGTA-3’
*WUSb*F	5’-CTTGCTTCGAGTCCGAACTACTC-3’
*WUSb*R	5’- AATTCGGTCGATCGGCGGATAC-3’
*WUSd*F	5’- CGATCCATACTTACTAGTAGCCG-3’
*WUSd*R	5’-ACATGGCGTTGGTGTTGCTGGCA-3’
*WOX2a*F	5’-CCGGCAGGACTTCTAGCATCATC-3’
*WOX2a*R	5’-CTATGGGCAAAGAAACAAATCCG-3’
*WOX2b*F	5’-CTGTCTTTCGCCGCTCTTGCCGT-3’
*WOX2b*R	5’-GCTTGTGAGTTGTGACCACCACG-3’
*WOX2d*F	5’- GTTTCCCTGCCTTCCGTCAAAG-3’
*WOX2d*R	5’-GAGATACTATGGCCAAACCCTC-3’
*WOX6a*F	5’- GTCGGGTTCGGTCTGGGTC-3’
*WOX6a*R	5’-TGGCTGCCTGCCTGCCTGGC-3’
*WOX6b*F	5’-GTCTTCCTTGAGATATAGTTCAGC-3’
*WOX6b*R	5’-CATGCATGTTCACTATAAACGTTC-3’
*WOX6d*F	5’-GGAGGAGCGCCGGAGCAGC-3’
*WOX6d*R	5’-AGCCGAGGAGGCTGCTGCT-3’
